# Exercise during pregnancy and infant body mass index during the first year of life: a secondary per-protocol analysis of a randomized clinical trial

**DOI:** 10.3389/fgwh.2026.1841700

**Published:** 2026-05-28

**Authors:** Rubén Barakat, Miguel Sánchez-Polán, Dingfeng Zhang, Maia Brik, Ignacio Refoyo, Ángeles Díaz-Blanco, Paloma Hernando, Iciar Olabarrieta, María Perales Santaella, Michelle F. Mottola

**Affiliations:** 1 AFIPE Research Group, Faculty of Physical Activity and Sport Sciences-INEF, Universidad Politécnica de Madrid, Madrid, Spain; 2 GICAF Research Group, Department of Education, Research and Evaluation Methods, Universidad Pontificia Comillas, Madrid, Spain; 3 School of Physical Education and Sports Science, South China Normal University, Guangzhou, China; 4 Obstetrics & Gynecology Department, Hospital Universitario Vall de Hebrón, Barcelona, Spain; 5 Sports Department, Faculty of Physical Activity and Sport Sciences-INEF, Universidad Politécnica de Madrid, Madrid, Spain; 6 Obstetrics & Gynecology Department, Hospital Universitario Severo Ochoa, Madrid, Spain; 7 Obstetrics & Gynecology Department, Hospital Universitario Puerta de Hierro de Majadahonda, Madrid, Spain; 8 Pediatrics Department, Hospital Universitario Severo Ochoa, Madrid, Spain; 9 Faculty of Health Sciences-HM Hospitals, Camilo Jose Cela University, Villanueva de la Cañada, Spain; 10 HM Hospitals Health Research Institute, Madrid, Spain; 11 Research Institute of Hospital 12 de Octubre (‘I+12’), Madrid, Spain; 12 R. Samuel McLaughlin Foundation-Exercise and Pregnancy Lab, School of Kinesiology, Faculty of Health Sciences, Department of Anatomy & Cell Biology, Schulich School of Medicine & Dentistry, Children’s Health Research Institute, The University of Western Ontario London, London, ON, Canada

**Keywords:** body mass index, children, exercise, physical activity, pregnancy

## Abstract

**Introduction:**

Maternal lifestyle during pregnancy may influence infant growth trajectories. Prenatal exercise improves maternal outcomes, but associations with infant Body Mass Index (BMI) beyond early infancy remain uncertain. The aim was to examine the association between participation in a supervised prenatal exercise program and infant BMI during the first year of life.

**Methods:**

This secondary per-protocol analysis was derived from a randomized clinical trial (NCT04563065) conducted in three public hospitals. Pregnant women were allocated to a supervised exercise program performed throughout pregnancy (IG = Intervention Group) or to standard obstetric care (CG = Control Group). Infant weight, length, BMI, and feeding type were assessed at 1, 2, 4, 6, and 12 months postpartum. Primary analyses followed a per-protocol approach restricted to mother–infant dyads meeting predefined adherence and follow-up criteria, with additional intention-to-treat sensitivity analyses.

**Results:**

Among 229 randomized participants, 126 mother–infant dyads were included in the per-protocol analyses. Infant BMI trajectories were similar between groups through 6 months of age. At 12 months, mean infant BMI was lower in the IG compared with controls in both per-protocol (16.49 ± 1.26 vs. 17.10 ± 1.40; *p* = 0.006) and intention-to-treat analyses (16.50 ± 1.25 vs. 17.07 ± 1.37; *p* = 0.007), although the absolute difference was modest. Infants in the CG had higher odds of overweight during mid and late infancy across both analytic approaches. Mothers in the IG gained less weight during pregnancy and had higher rates of exclusive breastfeeding during early infancy.

**Discussion:**

In this secondary per-protocol analysis, participation in a supervised prenatal exercise program was associated with lower infant BMI at 12 months and more favorable early feeding patterns. Given substantial attrition and the per-protocol analytic approach, findings should be interpreted cautiously and considered exploratory and hypothesis-generating.

## 1 Introduction

Pregnancy represents a critical period influencing long-term metabolic health in both mother and offspring, with potential implications for the development of obesity and related cardiometabolic conditions later in life (1).

From an epigenetic perspective, the intrauterine environment (whether beneficial or adverse) may have lasting effects beyond birth, with metabolic health being particularly sensitive to these early exposures. Emerging evidence highlights the increasing prevalence of childhood overweight and obesity as a key outcome associated with metabolic disturbances during pregnancy. In this context, conditions such as excessive gestational weight gain, pre-pregnancy overweight or obesity, and gestational diabetes have been consistently associated with adverse perinatal outcomes, including macrosomia, as well as with a higher risk of overweight and obesity in childhood (2–4).

The consequences of childhood overweight and obesity are multifactorial, extending beyond physiological effects to include significant psychological and emotional challenges. Notably, chronic conditions once predominantly observed in adults are now increasingly diagnosed in children with obesity (5, 6).

To mitigate these risks, numerous international organizations have recommended the promotion of physical activity during pregnancy as a preventive strategy (7–9). However, there remains a need for well-designed experimental studies to determine the optimal type, intensity, frequency, and duration of exercise throughout pregnancy to maximize these benefits (10). Such interventions may contribute to improving the intrauterine environment and reducing the risk of adverse health outcomes in the offspring (11–16).

This study presents a secondary analysis of a randomized controlled trial evaluating the effects of a supervised exercise program throughout pregnancy on birth weight and BMI at one year of age in the offspring. We hypothesize that structured prenatal exercise may influence birth weight and early growth trajectories during the first year of life.

## 2 Methods

### 2.1 Study design

Details of the original trial design and maternal outcomes have been reported previously and registered at ClinicalTrials.gov (NCT04563065). The study was conducted by the Universidad Politécnica de Madrid (UPM) in collaboration with Hospital Universitario Severo Ochoa, Hospital Universitario Puerta de Hierro, and Hospital Universitario Vall d'Hebrón. Ethical approval was obtained from the Research Ethics Committee (UPM). A detailed study protocol approved by the institutional ethics committee is available upon request.

### 2.2 Equity, diversity, and inclusion (EDI) statement

All pregnant women receiving routine care at our collaborating center and without contraindications for physical exercise were considered eligible for participation. Our multidisciplinary research team, comprising nine professionals in gynecology, obstetrics, midwifery, and exercise science, represents a breadth of expertise and diverse international perspectives. Team members work across different countries and healthcare systems, enhancing our study approach with varied insights and experiences.

Our team includes seven women and two men, collectively bringing specialized knowledge in obstetrics, gynecology, physical education, and research. We ensured no participant exclusion based on sociodemographic characteristics, and we maintained a focus on healthy pregnant women regardless of ethnicity, economic status, or educational background. Our commitment to equity, diversity, and inclusion aims to ensure that this research is both inclusive and representative of the diverse populations we serve.

### 2.3 Participants and randomization

Spanish-speaking pregnant women residing in Madrid were recruited and screened for eligibility during routine hospital obstetric visits (week 8–10 of pregnancy, see Study Algorithm). Participants were recruited from three hospitals within the same public healthcare system, serving comparable urban populations. Information about recruitment numbers per hospital is provided in Supplementary Table 2. Inclusion criteria were: age 19–48 years, no history or risk of preterm delivery, absence of multiple gestation and absence of pregnancy complications contraindicating exercise, and no participation in other trials or exercise programs. Exclusion criteria included plans to deliver at a different hospital, lack of continuous medical follow-up during pregnancy, or the presence of medical contraindications to safe exercise (8, 17). Informed written consent was obtained from all participants prior to enrollment. Participant enrollment occurred between August 5, 2020 and November 30, 2023.

The randomization process was managed using RedCap software (18), with participants randomly assigned to the Intervention Group (IG) or Control Group (CG) in a 1:1 ratio. A computer-generated randomization sequence was securely uploaded to the RedCap database, with one healthcare provider at each hospital managing access to maintain confidentiality of group assignments from other hospital staff.

### 2.4 Intervention

The exercise program consisted of three weekly sessions, each lasting 60 min, incorporating complementary and interrelated activities divided into seven structured parts. The distribution of program components was dynamically adjusted across pregnancy stages to address the evolving physical needs and adaptations associated with gestation (19) (Table 1).

**Table 1:** Percentage distribution of exercise components across pregnancy stages in the intervention program.

Components	Until week 20	Until week 30	Until weeks 38–39
Aerobic Endurance %	40	30	25
Muscle Strengthening %	30	25	25
Coordination/Postural Equilibrium (Balance) %	10	15	15
Stretching/Relaxation %	10	15	15
Pelvic Floor Muscle Training %	10	15	20

Data are presented as percentages of total session time allocated to each exercise component.

A minimum adherence threshold of 70% was predefined, corresponding to approximately 60 supervised sessions across pregnancy. Attendance was recorded using a dedicated computer application. Among participants included in the per-protocol analysis, mean adherence was 86.2%, corresponding to approximately 75 supervised sessions, or an average of approximately 2.3 sessions per week across gestation. This adherence rate is comparable to rates reported in previous studies utilizing the same model (19), but exclusively through in-person sessions (20).

Exercise intensity was managed by maintaining participants' heart rates within 55%–65% of their maximum maternal heart rate, calculated using the Karvonen formula (21), alongside a perceived exertion level of 12–14 (“Somewhat Hard”) on Borg's Rating of Perceived Exertion Scale (22).

The CG received standard obstetric care, comparable to that provided to the IG. Participants were given informational materials covering urinary incontinence, physical activity, sleep hygiene, smoking cessation, and nutritional guidelines; however, no supervised exercise sessions were conducted as part of the program for this group. While CG participants were not discouraged from engaging in independent exercise, their physical activity levels were monitored once per trimester using a “Decision Algorithm” administered via telephone (23).

### 2.5 Outcomes

Data acquisition was facilitated by the SELENE platform, which encompassed obstetric records for the mother, fetus, and newborn before, during, and after pregnancy. The primary completion date registered corresponds to completion of maternal pregnancy outcomes. Infant follow-up up to 12 months postpartum was conducted as an extended follow-up.

BMI at 1, 2, 4, 6, and 12 months postpartum was the primary focus of the present secondary analysis and was not a registered primary outcome of the original trial. BMI was calculated as weight (kg) divided by length squared (m^2^), and age- and sex-adjusted BMI *Z*-scores and percentiles were derived accordingly (24, 25). The BMI percentile cut-off points according to BMI *Z*-scores are presented as Supplementary Table 1.

Infant follow-up visits were scheduled at 1, 2, 4, 6, and 12 months postpartum. Infant weight and length were measured using standardized procedures, BMI was calculated, and feeding type was recorded at each visit. For postnatal analyses, infants born before 34 weeks of gestation were excluded. Maternal height and pre-pregnancy weight were collected at baseline, maternal weight at delivery, and exercise exposure during pregnancy was monitored through supervised session attendance. Postpartum maternal exercise behavior was not systematically assessed.

Secondary outcomes included pregestational descriptive and perinatal information. Pregestational descriptive data comprised maternal age, parity, previous miscarriage, occupation, ethnicity, pregestational weight, height, and BMI before and during pregnancy. Maternal BMI was calculated as weight (kg) divided by height squared (m^2^), with classifications as underweight (BMI <18.5 kg/m^2^), normal weight (BMI 18.5–24.9 kg/m^2^), overweight (BMI 25.0–29.9 kg/m^2^), and obese (BMI ≥30 kg/m^2^) (26).

Perinatal data included gestational age, adherence to the exercise program, occurrence of gestational diabetes, total gestational weight gain (kg) and occurrence of excessive maternal weight gain. Total gestational weight gain was assessed as the difference between pre-pregnancy weight and weight at delivery. Occurrence of excessive maternal weight gain was classified according to the 2009 Institute of Medicine (IOM) guidelines according to the following pre-pregnancy BMI categories: >18 kg for underweight participants, >16 kg for normal weight participants, >11.5 kg for overweight participants, and >9 kg for obese participants (27).

Delivery information included mode of delivery (vaginal or cesarean section), type of delivery (preterm <37 weeks gestation), infant sex, birth weight, length, BMI, head circumference, umbilical cord blood pH, Apgar scores at 1 and 5 min, and neonatal intensive care unit (NICU) admissions. Birth weight classifications were as follows: low birth weight (<2,500 g), adequate birth weight (2,500–4,000 g), and macrosomia (>4,000 g) (28). Adjusted for gestational age at delivery, weight below the 10th percentile was classified as small for gestational age (SGA), between the 10th and 90th percentiles as appropriate for gestational age (AGA), and above the 90th percentile as large for gestational age (LGA). Postpartum data included type of feeding (exclusive breastfeeding, formula feeding, or mixed).

### 2.6 Sample size calculation

The original sample size calculation for the randomized trial was based on maternal pregnancy outcomes, particularly gestational weight gain, as defined in the trial protocol. Infant growth outcomes were not included in the original sample size calculation. Descriptive assumptions regarding childhood overweight are provided for contextual reference only. Accordingly, no formal sample size calculation was performed for infant BMI outcomes in this secondary analysis.

### 2.7 Statistical analysis

Statistical analysis and presentation are consistent with the CHAMP statement (29).

Primary analyses were conducted using a per-protocol approach, restricted to participants meeting predefined adherence and follow-up criteria. In addition, intention-to-treat (ITT) analyses were performed as a sensitivity analysis, including all randomized participants with available outcome data at each time point.

No imputation of missing data was applied; therefore, ITT analyses were based on available cases. The consistency between per-protocol and ITT findings was examined to assess the robustness of the results. Given the secondary and exploratory nature of the infant growth outcomes, no formal adjustment for multiple comparisons was applied. Therefore, *p*-values should be interpreted cautiously and considered exploratory rather than confirmatory.

Statistical analyses were conducted to compare maternal and infant categorical and continuous variables between groups using appropriate parametric (independent *t*-tests) and non-parametric tests, as applicable.

For categorical outcomes, multinomial logistic regression models were used to estimate associations between group allocation and infant BMI categories at each follow-up time point, using normal weight as the reference category.

Continuous variables are presented as means and standard deviations, and categorical variables as frequencies and percentages. Effect estimates are reported as mean differences and odds ratios with 95% confidence intervals. Statistical significance was set at *p* < 0.05.

## 3 Results

The following findings were not adjusted for multiple comparisons and should therefore be interpreted as exploratory.

### 3.1 Study participant recruitment and retention

**Figure 1:**
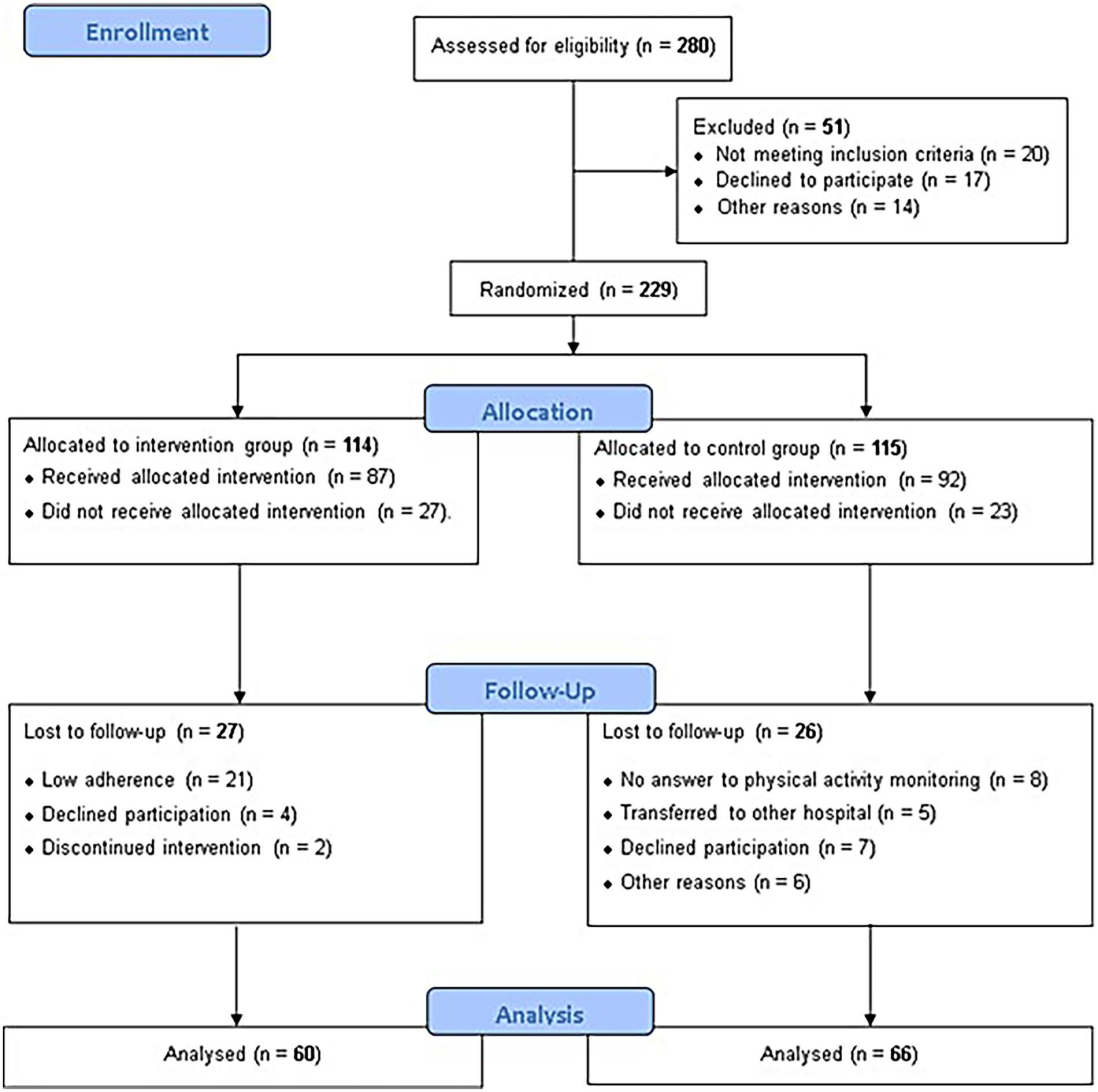
Flow chart of study participants.

A total of 280 women were screened for eligibility according to the trial protocol. After exclusion of 51 women, 229 were randomized to the IG or the CG. Following allocation and subsequent follow-up, 60 women in the IG and 66 women in the CG completed all infant evaluations and met predefined per-protocol criteria, and were included in the final analytic sample (Figure 1).

### 3.2 Maternal characteristics at baseline

Table 2 shows the characteristics of the participants at the beginning of the study in both groups, with no statistically significant differences between groups at baseline (*p* > 0.05).

**Table 2:** Baseline characteristics of participants included in the study.

Outcomes/analysis	Intention to Treat		*p*-value	Per Protocol		*p*-value
	IG (*n* = 114)	CG (*n* = 115)		IG (*n* = 60)	CG (*n* = 66)	
Age (y)	35.37 ± 4.27	34.46 ± 4.87	0.135	36.02 ± 4.18	35.00 ± 5.38	0.242
Weight (kg)	68.51 ± 16.87	65.86 ± 15.17	0.235	63.77 ± 11.10	65.15 ± 15.16	0.588
Height (cm)	162.75 ± 6.01	163.33 ± 6.99	0.510	162.91 ± 5.76	162.95 ± 7.32	0.968
BMI	25.78 ± 5.66	24.75 ± 5.60	0.188	24.05 ± 3.82	24.74 ± 5.92	0.462
Categories of BMI (*n*/%)						
Underweight	0/0.0%	4/3.8%	0.037*	0/0.0%	4/6.9%	0.164
Normal weight	53/51.5%	64/60.4%		35/64.8%	31/53.4%	
Overweight	31/30.1%	18/17%		13/24.1%	13/22.4%	
Obese	19/18.4%	20/18.9%		6/11.1%	10/17.2%	
Ethnicity (*n*/%)						
Caucasian	97/91.5%	101/88.6%	0.543	55/94.8%	61/93.8%	0.633
Latin American	9/8.5%	12/10.5%		3/5.2%	3/4.6%	
Afro American	0/0%	1/0.9%		0/0%	1/1.5%	
Occupation (*n*/%)						
House maker	6/5.8%	6/5.6%	0.433	4/6.9%	4/6.5%	0.413
Passive job	68/66%	62/57.9%		39/67.2%	35/56.5%	
Active job	29/28.2%	39/36.4%		15/25.9%	23/37.1%	
Parity (*n*/%)						
Nulliparous	73/67.6%	78/69%	0.947	40/69%	46/70.8%	0.375
One	26/24.1%	26/23%		15/25.9%	12/18.5%	
Two or more	9/8.3%	9/8%		3/5.2%	7/10.8%	
Previous miscarriage						
None	74/69.2%	80/71.4%	0.710	44/75.9%	41/63.1%	0.304
One	29/27.1%	26/23.2%		12/20.7%	20/30.8%	
Two or more	4/3.7%	6/5.4%		2/3.4%	4/6.2%	

* Results should be interpreted with caution due to the small number of observations in some categories.

### 3.3 Newborn and infant outcomes from birth until month 12

Infant birth outcomes did not differ between groups (Table 3). Birth weight, birth length, head circumference, Apgar scores, umbilical cord pH, and distribution across weight-for-gestational-age categories were similar in the IG and the CG.

**Table 3:** Infant outcomes from birth until 12 months.

Outcomes/Analysis	Intention to Treat		*p*-value	Per Protocol		*p*-value
	IG (*n* = 99)	CG (*n* = 100)		IG (*n* = 60)	CG (*n* = 66)	
Gender (*n*/%)						
Male	36/48.6%	47/53.4%	0.546	29/49.2%	34/51.5%	0.792
Female	38/51.4%	41/46.6%		30/50.8%	32/48.5%	
NICU Admission (*n*/%)						
No	63/82.9%	69/80.2%	0.663	47/78.3%	56/84.8%	0.344
Yes	13/17.1%	17/19.8%		13/21.7%	10/15.2%	
Weight for gestational age (*n*/%)						
SGA	16/21.6%	17/18.5%	0.544	12/20.3%	12/19.4%	0.945
AGA	54/73%	66/71.7%		44/74.6%	46/74.1%	
LGA	4/5.4%	9/9.8%		3/5.1%	4/6.5%	
Head circumference (cm)	34.34 ± 1.39	34.64 ± 1.30	0.062	34.33 ± 1.48	34.54 ± 1.21	0.189
Birth length (cm)	49.55 ± 2.25	49.64 ± 2.08	0.375	49.38 ± 2.42	49.50 ± 2.06	0.386
Birth weight (g)	3,165.39 ± 487.10	3,223.21 ± 517.26	0.209	3,090.67 ± 501.11	3,177.32 ± 505.44	0.168
Umbilical cord pH	7.25 ± 0.076	7.23 ± 0.082	0.198	7.24 ± 0.076	7.24 ± 0.083	0.772
Apgar 1 min	8.79 ± 0.713	8.71 ± 0.810	0.240	8.73 ± 0.842	8.69 ± 0.767	0.394
Apgar 5 min	9.84 ± 0.421	9.80 ± 0.481	0.277	9.82 ± 0.471	9.76 ± 0.508	0.268
Birth BMI	12.70 ± 1.24	12.99 ± 1.36	0.077	12.60 ± 1.27	12.89 ± 1.27	0.100
1 month BMI	13.53 ± 1.45	13.93 ± 1.54	0.062	13.54 ± 1.47	13.93 ± 1.56	0.075
2 months BMI	15.10 ± 1.34	15.53 ± 1.67	**0** **.** 045*	15.07 ± 1.33	15.49 ± 1.66	0.063
4 months BMI	16.28 ± 1.63	16.56 ± 1.85	0.174	16.25 ± 1.65	16.57 ± 1.92	0.155
6 months BMI	16.77 ± 1.41	17.04 ± 1.75	0.161	16.78 ± 1.46	17.05 ± 1.81	0.182
12 months BMI	16.50 ± 1.25	17.07 ± 1.37	**0** **.** 007*	16.49 ± 1.26	17.10 ± 1.40	**0** **.** 006*

Data are expressed as mean ± standard deviation or sample size/percentage. IG, Intervention Group; CG, Control Group; NICU, The Neonatal Intensive Care Unit; SGA, Small for Gestational Age; AGA, Appropriate for Gestational Age; LGA, Large for Gestational Age; BMI, Body Mass Index.

*Results should be interpreted with caution due to the small number of observations in some categories.

Bold text: *p* = <0.05.

Infant BMI trajectories were comparable from birth through 6 months. However, at 12 months, infants in the IG had a significantly lower mean BMI compared with those in the CG (16.49 ± 1.26 vs. 17.10 ± 1.40; *p* = 0.006). No group differences were observed in NICU admission rates or sex distribution.

### 3.4 Classification of BMI from birth until 12 months

BMI categories at each follow-up point are shown in Table 4. At 4 months, infants in the CG had higher odds of being classified as overweight (OR: 6.44; 95% CI: 1.38–30.13; *p* = 0.008). This difference persisted at 12 months, with higher odds of overweight in the CG (OR: 3.03; 95% CI: 1.23–7.51; *p* = 0.014). No significant differences were observed for underweight or normal-weight classifications at any time-point.

**Table 4:** Distribution of infant BMI categories and multinomial logistic regression analysis by study group and follow-up time point.

Outcome	Timepoint	IG	CG	OR (95% CI)	*p*-value
Birth measure	Underweight (ITT)	5/6.8%	8/9.3%	1.42 (.44, 4.53)	0.557
	Normal weight (ITT)	67/90.5%	75/87.2%	.71 (.26, 1.94)	0.506
	Overweight (ITT)	2/2.7%	3/3.5%	1.30 (.21, 8.01)	0.776
	Underweight (PP)	5/8.3%	6/9.1%	1.10 (.32, 3.81)	0.880
	Normal weight (PP)	53/88.3%	59/89.4%	1.11 (.37, 3.38)	0.850
	Overweight (PP)	2/3.3%	1/1.5%	.45 (.04, 5.05)	0.504
1st month	Underweight (ITT)	28/44.4%	30/40%	.83 (.42, 1.64)	0.598
	Normal weight (ITT)	33/52.4%	41/55.4%	1.13 (.58, 2.22)	0.723
	Overweight (ITT)	2/3.2%	4/5.5%	1.77 (.31, 9.99)	0.514
	Underweight (PP)	26/43.3%	26/39.4%	.85 (.42, 1.73)	0.654
	Normal weight (PP)	32/53.3%	36/54.5%	1.05 (.52, 2.12)	0.892
	Overweight (PP)	2/3.3%	4/6.1%	1.87 (.33, 10.60)	0.473
2nd month	Underweight (ITT)	24/36.9%	21/28%	.66 (.33, 1.36)	0.260
	Normal weight (ITT)	37/56.9%	46/61.3%	1.2 (.61, 2.36)	0.596
	Overweight (ITT)	4/6.2%	8/10.7%	1.82 (.25, 6.35)	0.341
	Underweight (PP)	22/36.7%	18/27.3%	.65 (.31, 1.38)	0.258
	Normal weight (PP)	35/58.3%	42/63.6%	1.25 (.61, 2.56)	0.542
	Overweight (PP)	3/5%	6/9.1%	1.90 (.45, 7.96)	0.373
4th month	Underweight (ITT)	15/23.4%	19/25.7%	1.13 (.52, 2.46)	0.761
	Normal weight (ITT)	47/73.4%	43/58.1%	.50 (.24, 1.03)	0.059
	Overweight (ITT)	2/3.1%	12/16.2%	6.0 (1.29, 27.93)	**0** **.** **011** *
	Underweight (PP)	15/25%	17/25.8%	1.04 (.47, 2.33)	0.922
	Normal weight (PP)	43/71.7%	37/56.1%	.50 (.24, 1.06)	0.069
	Overweight (PP)	2/3.3%	12/18.2%	6.44 (1.38, 30.13)	**0** **.** **008** *
6th month	Underweight (ITT)	13/19.7%	17/22.7%	1.20 (.53, 2.69)	0.667
	Normal weight (ITT)	48/72.7%	46/61.3%	.60 (.29, 1.21)	0.152
	Overweight (ITT)	5/7.6%	12/16%	2.32 (.77, 6.99)	0.125
	Underweight (PP)	12/20.3%	15/22.7%	1.15 (.49, 2.71)	0.746
	Normal weight (PP)	42/71.2%	39/59.1%	.59 (.28, 1.23)	0.157
	Overweight (PP)	5/8.5%	12/18.2%	2.40 (.79, 7.28)	0.114
1st year	Underweight (ITT)	9/14.1%	5/6.7%	.44 (.14, 1.37)	0.149
	Normal weight (ITT)	47/73.4%	48/64%	.64 (.31, 1.33)	0.233
	Overweight (ITT)	8/12.5%	22/29.3%	2.91 (1.19, 7.09)	**0** **.** **016** *
	Underweight (PP)	9/15%	4/6.1%	.37 (.11, 1.26)	0.099
	Normal weight (PP)	43/71.7%	41/62.1%	.65 (.31, 1.37)	0.256
	Overweight (PP)	8/13.3%	21/31.8%	3.03 (1.23, 7.51)	**0** **.** **014** *

Data are expressed as sample size/percentage. IG, Intervention Group; CG, Control Group; OR, Odds Ratio; CI, Confidence Interval; ITT, Intention to Treat; PP, Per Protocol.

* Results should be interpreted with caution due to the small number of observations in some categories.

Bold text: *p* = <0.05.

### 3.5 Maternal outcomes

Maternal outcomes are summarized in Table 5. Women in the IG gained significantly less weight during pregnancy compared with the CG (7.71 ± 5.20 vs. 9.74 ± 6.10 kg; *p* = 0.035). Although the proportion of excessive gestational weight gain was lower in the IG, this did not reach statistical significance (*p* = 0.063). Type of delivery, maternal BMI categories at delivery, BMI at delivery, Gestational Diabetes and gestational age at birth did not differ significantly between groups.

**Table 5:** Maternal outcomes at the end of pregnancy.

Outcomes/analysis	Intention to treat		*p*-value	Per protocol		*p*-value
	IG (*n* = 101)	CG (*n* = 103)		IG (*n* = 58)	CG (*n* = 63)	
Excessive weight gain (*n*/%)						
No	81/84.4%	76/80%	0.429	47/90.4%	40/76.9%	0.063
Yes	15/15.6%	19/20%		5/9.6%	12/23.1%	
Type of delivery (*n*/%)						
Spontaneous vaginal delivery	54/55.1%	55/56.1%	0.223	31/53.4%	31/49.2%	0.257
Instrumental	25/25.5%	32/32.7%		14/24.1%	23/36.5%	
Cesarean section	19/19.4%	11/11.2%		13/22.4%	9/14.3%	
Categories of BMI (*n*/%)						
Normal weight	21/21.2%	30/29.4%	0.230	17/30.9%	19/32.8%	0.190
Overweight	49/49.5%	39/38.2%		28/50.9%	21/36.2%	
Obese	29/29.3%	33/32.4%		10/18.2%	18/31%	
BMI at delivery	28.85 ± 5.37	28.45 ± 5.54	0.301	26.93 ± 3.28	28.28 ± 5.60	0.130
Weight at delivery (kg)	76.52 ± 16.03	75.77 ± 15.01	0.366	71.57 ± 10.23	74.85 ± 14.57	0.060
Weight gain (kg)	8.95 ± 5.48	9.67 ± 5.49	0.181	7.71 ± 5.20	9.74 ± 6.10	0.035*
Gestational week at delivery (w)	39.23 ± 1.55	39.16 ± 1.52	0.387	39.23 ± 1.61	39.09 ± 1.49	0.606

Data are presented as mean ± SD or *n* (%). Group comparisons were performed using independent t-tests for continuous variables and chi-square tests for categorical variables.

*Results should be interpreted with caution due to the small number of observations in some categories.

### 3.6 Breastfeeding type during the first year

Breastfeeding patterns differed significantly between groups from 1 to 6 months (Table 6). Exclusive breastfeeding rates were consistently higher in the IG at 1, 2, 4, and 6 months (71.2% vs. 45.2%; *p* = 0.015). Formula feeding was more frequent in the CG at each of these intervals. At 12 months, breastfeeding type did not differ significantly between groups. Interaction terms between group allocation and infant feeding type were tested; no statistically significant interactions were observed.

**Table 6:** Breastfeeding type during the first year.

Outcomes/Analysis	Intention to Treat		*p*-value	Per Protocol		*p*-value
	IG (*n* = 66)	CG (*n* = 79)		IG (*n* = 60)	CG (*n* = 66)	
Breastfeeding type 1 month (*n*/%)						
Exclusive breastfeeding	59/92.2%	52/65.8%	<0.001*	55/91.7%	43/65.2%	<0.001*
Formula feeding	0/0%	14/17.7%		0/0%	12/18.2%	
Mixed feeding	5/7.8%	13/16.5%		5/8.3%	11/16.6%	
Breastfeeding type 2 months (*n*/%)						
Exclusive breastfeeding	56/84.8%	45/57%	**0** **.** 001*	51/85%	35/53.8%	<0.001*
Formula feeding	4/6.1%	18/22.8%		4/6.7%	16/24.6%	
Mixed feeding	6/9.1%	16/20.3%		5/8.3%	14/21.5%	
Breastfeeding type 4 months (*n*/%)						
Exclusive breastfeeding	51/78.5%	41/55.4%	**0** **.** 016*	46/78%	32/50.8%	**0** **.** 008*
Formula feeding	8/12.3%	20/27%		8/13.5%	20/31.7%	
Mixed feeding	6/9.2%	13/17.6%		5/8.5%	11/17.5%	
Breastfeeding type 6 months (*n*/%)						
Exclusive breastfeeding	47/71.2%	37/50.7%	**0** **.** 046*	42/71.2%	28/45.2%	**0** **.** 015*
Formula feeding	12/18.2%	24/32.9%		12/20.3%	24/38.7%	
Mixed feeding	7/10.6%	12/16.4%		5/8.5%	10/16.1%	
Breastfeeding type 12 months (*n*/%)						
Exclusive breastfeeding	31/48.4%	27/38.6%	0.466	27/46.6%	20/33.3%	0.317
Formula feeding	28/43.8%	38/54.3%		27/46.6%	36/60%	
Mixed feeding	5/7.8%	5/7.1%		4/6.9%	4/6.7%	

Data are presented as *n* (%). Group comparisons were performed using chi-square tests. IG, intervention group; CG, control group.

*Results should be interpreted with caution due to the small number of observations in some categories.

Bold text: *p* = <0.05.

### 3.7 Sensitivity analyses (intention-to-treat)

Sensitivity analyses using an intention-to-treat approach yielded results consistent with the per-protocol analyses. In particular, lower mean BMI values were observed in the intervention group at 12 months (16.50 ± 1.25 vs. 17.07 ± 1.37; *p* = 0.007), mirroring the per-protocol findings. Similarly, the odds of overweight remained higher in the control group at 4 and 12 months. Although effect sizes were slightly attenuated, the direction and statistical significance of key outcomes were preserved across analytic approaches.

These findings suggest that the main results are directionally robust but sensitive to adherence and completeness of follow-up.

## 4 Discussion

In this secondary per-protocol analysis of a randomized clinical trial, participation in a structured prenatal exercise intervention was associated with differences in infant growth patterns during the first year of life. While neonatal anthropometric measures did not differ between groups, infants in the intervention group exhibited lower BMI at 12 months and a lower prevalence of overweight at 4 and 12 months compared with controls. These findings align with prior evidence suggesting that maternal lifestyle behaviors during pregnancy, particularly physical activity, may be associated with offspring growth trajectories and early obesity risk (30).

Sensitivity analyses based on an intention-to-treat approach yielded results consistent in direction with the per-protocol analyses, although with attenuated effect sizes. This pattern is consistent with methodological expectations, as per-protocol analyses may overestimate intervention effects due to selection based on adherence. Importantly, the consistency of findings across both per-protocol and intention-to-treat analyses strengthens the robustness of the observed associations, reducing the likelihood that results are solely driven by adherence-related selection bias (31). The consistency between approaches provides some reassurance regarding the robustness of the findings, while also highlighting the influence of adherence and follow-up on observed outcomes.

Although the absolute difference in mean BMI at 12 months was modest (∼0.6 kg/m^2^), its clinical interpretation warrants consideration beyond central tendency. Small shifts in mean BMI at the population level may reflect more meaningful changes in the distribution of weight status, particularly at the upper end of the spectrum (32). In the present study, this is supported by the higher odds of overweight observed in the control group at both 4 and 12 months.

From a clinical perspective, infant growth assessment is typically based on WHO BMI-for-age percentiles rather than absolute BMI values. Therefore, even modest differences in BMI may correspond to shifts across percentile thresholds, which are more directly linked to clinical risk stratification. In this context, the observed findings may suggest an early divergence in growth trajectories, although their long-term clinical significance remains uncertain.

A key consideration in interpreting these findings is the limited temporal scope of the follow-up. Infant BMI during the first year of life is characterized by rapid and dynamic changes in growth and body composition, and its predictive value for later obesity is relatively limited. Therefore, the observed differences at 12 months should not be interpreted as evidence of sustained effects or long-term obesity prevention (33).

Rather, these findings may reflect early divergence in growth trajectories that could be influenced by prenatal and early postnatal factors. Whether such differences persist, attenuate, or amplify over time remains unknown. Longer-term follow-up into early childhood and school age is required to determine the extent to which these early-life differences translate into clinically meaningful outcomes.

A particularly relevant finding in the present study is the higher rate of exclusive breastfeeding observed among women in the intervention group during the first six months postpartum. Exclusive breastfeeding is strongly recommended by WHO and UNICEF and has been consistently associated with more favorable infant growth patterns and a reduced risk of later overweight and obesity (34).

In this context, breastfeeding may represent a key behavioral pathway linking prenatal exercise to infant outcomes. Maternal participation in structured exercise programs during pregnancy may reflect or promote broader health-oriented behaviors, including increased likelihood of breastfeeding initiation and continuation (35). This behavioral clustering is supported by emerging evidence suggesting synergistic effects of maternal physical activity and breastfeeding on infant metabolic health.

Although formal mediation analyses were not performed, the observed differences in breastfeeding patterns may partially explain the more favorable BMI profile observed in the intervention group. This interpretation provides a clinically meaningful framework in which prenatal exercise interventions may contribute to infant health not only through direct physiological mechanisms but also by promoting beneficial postnatal behaviors.

In addition to breastfeeding, other postnatal factors may have contributed to the observed differences in infant BMI at 12 months. The introduction of complementary feeding, dietary composition, feeding practices, and broader family lifestyle behaviors are known to play an important role in shaping infant growth trajectories during the second half of the first year of life.

As these variables were not systematically assessed in the present study, their potential influence cannot be excluded. It is therefore likely that the observed differences reflect the combined effect of prenatal influences and postnatal environmental factors, rather than the isolated effect of the exercise intervention.

Maternal outcomes further contextualize the infant findings. Women in the intervention group experienced lower gestational weight gain, consistent with meta-analytic evidence demonstrating that prenatal lifestyle interventions reduce gestational weight gain and improve maternal health (36). Given the well-established association between excessive gestational weight gain and childhood adiposity (36), the concurrent observation of lower maternal weight gain and more favorable infant growth patterns supports a biologically coherent, though non-causal, framework linking maternal and infant outcomes.

Mode of delivery did not differ between groups, reducing the likelihood that delivery mode confounded the observed infant growth differences. This finding is consistent with existing evidence indicating that prenatal exercise does not increase the risk of cesarean delivery or intrapartum complications (37). The absence of differences in neonatal anthropometry also aligns with prior research showing that prenatal exercise does not typically influence birth size but may be associated with delayed effects on infant growth and metabolic regulation (30, 37).

The characteristics of the study population should also be considered when interpreting the findings. Participants were primarily healthy pregnant women without major obstetric or metabolic complications, which may limit the generalizability of the results to broader clinical populations. Therefore, the present findings are most directly applicable to low-risk pregnancies receiving standard obstetric care.

However, it is possible that the effects of prenatal exercise on maternal and infant outcomes may be more pronounced in higher-risk populations, such as women with pre-pregnancy obesity, excessive gestational weight gain, or gestational diabetes. These groups are characterized by greater metabolic vulnerability and may derive greater benefit from structured lifestyle interventions (38). Future studies specifically targeting high-risk populations are needed to evaluate the extent to which these findings can be extended to clinical settings where the burden of adverse outcomes is higher.

These findings support pregnancy as a critical window for preventive health interventions. Although causal inferences cannot be drawn from this secondary per-protocol analysis, the observed associations with maternal weight regulation, breastfeeding practices, and infant growth patterns are consistent with broader evidence suggesting potential benefits of prenatal physical activity. Structured and supervised exercise programs, widely endorsed as safe during pregnancy, may represent a valuable component of comprehensive antenatal health promotion strategies. Further adequately powered, prospectively registered trials are required to inform clinical implementation and guideline development. The clinical significance of the observed BMI differences remains uncertain and should be interpreted in light of their modest magnitude and early timing.

Further studies with extended follow-up are required to determine whether these early differences persist and translate into meaningful health outcomes.

### 4.1 Strengths and limitations

This study has several strengths. It is based on a randomized clinical trial with prospective data collection and includes longitudinal follow-up of infant growth during the first year of life. The integration of maternal pregnancy outcomes, infant anthropometry, and feeding patterns within a structured and supervised prenatal exercise intervention provides a comprehensive framework to explore early-life growth trajectories in a real-world public healthcare setting.

However, several limitations should be considered. First, the primary analyses were conducted using a per-protocol approach, restricted to participants meeting predefined adherence and follow-up criteria. This results in loss of randomization and introduces potential selection bias, limiting causal inference. Although intention-to-treat analyses were conducted as a sensitivity approach and showed consistent directional results, these were based on available cases and may still be affected by missing data. Furthermore, although ITT analyses were conducted, they were based on available cases without imputation, which may still introduce bias related to missing outcome data.

Second, attrition between randomization and final follow-up was substantial, a common challenge in longitudinal perinatal research and further impacted by the COVID-19 pandemic. This may reduce generalizability and influence the stability of estimates.

Third, infant growth outcomes were not prespecified primary endpoints of the original trial, and the sample size calculation was based on maternal outcomes. In addition, no formal adjustment for multiple comparisons was performed. Accordingly, all statistically significant findings should be interpreted cautiously and considered exploratory and hypothesis-generating.

Fourth, this manuscript represents a secondary analysis of a previously reported randomized trial. While maternal and perinatal outcomes have been published elsewhere, the present study focuses specifically on infant growth trajectories and does not duplicate primary outcomes.

Fifth, several relevant exposures were not comprehensively assessed, including complementary feeding practices, detailed infant dietary intake, postpartum maternal physical activity, and objective measures of maternal fitness or exercise intensity. These unmeasured factors may have contributed to the observed associations.

Sixth, the duration of follow-up was limited to the first year of life. Given the dynamic nature of infant growth and the limited predictive value of BMI in early infancy, the long-term clinical significance of the observed differences remains uncertain.

Finally, the study population consisted primarily of healthy, low-risk pregnant women, which may limit the generalizability of the findings to more diverse or higher-risk populations.

Taken together, these limitations indicate that the findings should be interpreted as exploratory and hypothesis-generating. Future studies with prospective registration, adequately powered intention-to-treat analyses, comprehensive exposure assessment, and longer-term follow-up are required to confirm these results and clarify their clinical relevance.

## Data Availability

The datasets presented in this article are not readily available because participant data will not be shared, due to it contain confidential and sensitive information for patients. Requests to access the datasets should be directed to participant data will not be shared, due to it contain confidential and sensitive information for patients.
