# Supplementary material for: Exercise during pregnancy and infant body mass index during the first year of life: a secondary per-protocol analysis of a randomized clinical trial
Source: Front Glob Womens Health. 2026 May 28;7:1841700. doi: 10.3389/fgwh.2026.1841700 (PMC13253496; doi:10.3389/fgwh.2026.1841700)
Supplement: Supplementary file 1 [file Table1.docx]

Supplementary Material

# Supplementary Tables

Supplementary Table 1. BMI percentile cut-off depending on BMI Z-scores based on infant sex.

| **Supplementary Table 1.** BMI percentile cut-off depending on BMI Z-scores based on infant sex. | | | | | | |
| --- | --- | --- | --- | --- | --- | --- |
| **Variable (Kg/m^2^)** | **Sex** | **Strong Underweight** | **Underweight** | **Normal Weight** | **Overweight** | **High Overweight Obesity** |
|  |  | Percentile <5th | | Percentile 5-85th | Percentile >85th | |
| BMI at 1 month | M | 10.0-12.3 | 12.3-13.5 | 13.5-16.2 | 16.2 -17.6 | >17.6 |
|  | F | 9.0-11.8 | 11.8-13.1 | 13.1-15.8 | 15.8-17.4 | >17.4 |
| BMI at 2 months | M | 11.0-13.6 | 13.6-14.8 | 14.8-17.6 | 17-6-19.2 | >19.2 |
|  | F | 10.0-12.9 | 12.9-14.2 | 14.2-17.2 | 17.2-18.8 | >18.8 |
| BMI at 4 months | M | 12.0-14.5 | 14.5-15.8 | 15.8-18.7 | 18.7-20.3 | >20.3 |
|  | F | 11.0-13.9 | 13.9-15.2 | 15.2-18.3 | 18.3-20.0 | >20.0 |
| BMI at 6 months | M | 12.0-14.7 | 14.7-16.0 | 16.0-18.8 | 18.8-20.5 | >20.5 |
|  | F | 12.0-14.1 | 14.1-15.5 | 15.5-18.5 | 18.5-20.2 | >20.3 |
| BMI at 12 months | M | 12.0-14.3 | 14.3-15.4 | 15.4-18.1 | 18.1-19.7 | >19.7 |
|  | F | 11.0-13.7 | 13.7-14.9 | 14.9-17.7 | 17.7-19.5 | >19.5 |

Supplementary Table 2. Hospital recruitment numbers.

| **Supplementary Table 2. Hospital recruitment numbers.** | |
| --- | --- |
| **Hospital** | **Recruitment pregnant**  **Assessed for eligibility (n= 280)** |
| Hospital Universitario Puerta de Hierro | 70 |
| Hospital Universitario Severo Ochoa | 145 |
| Hospital Universitario Vall d´Hebrón | 65 |
